# Tuberculosis in Undocumented Migrants, Geneva

**DOI:** 10.3201/eid1102.030215

**Published:** 2005-02

**Authors:** Sigiriya A. Perone, Patrick Bovier, Christian Pichonnaz, Thierry Rochat, Louis Loutan

**Affiliations:** * Geneva University Hospital, Geneva, Switzerland

**Keywords:** letter, tuberculosis, migrant, resistance

**To the Editor:** In today's globalized world, a growing number of people are migrating in search of a better life. Simultaneously, industrialized countries are strengthening border controls and administrative barriers to contain this influx of newcomers, resulting in a significant increase in illegal migration and human trafficking. The U.S. Department of State estimates the annual flow of irregular migrants worldwide to be 700,000–2 million (*1*). Many of the migrants are from countries where tuberculosis is endemic, and they contribute to the increasing proportion of foreign-born persons with tuberculosis in North America and Europe. These persons may be highly contagious in the local population, as they have limited access to healthcare and often go untreated (*2**,**3*).

Of 450,000 residents in Geneva, Switzerland, 10,000–20,000 are undocumented and come from developing countries or Eastern Europe. All patients treated for tuberculosis in Geneva are systematically registered by the Antituberculosis Center, a facility at Geneva University Hospital. An outpatient clinic provides free consultations for patients with tuberculosis who have no health insurance, and patients are not required to disclose their immigration status to physicians. Patients with sufficient funds pay for their medication.

All cases of tuberculosis in undocumented migrants (foreign residents with no resident permits) reported from 1994 to 1998 were reviewed by the same investigator. Their sociodemographic and clinical characteristics were compared with those of 7 South American legal residents with tuberculosis (representing the whole sample of South American tuberculosis patients) during the same period and with a group of 50 tuberculosis patients from the general population. Characteristics of HIV-positive and HIV-negative patients with tuberculosis were compared also.

From 1994 to 1998, a total of 397 persons in Geneva were notified that they were infected with tuberculosis. Twenty-two (6%) case-patients were found among undocumented migrants. The mean age was 31 years (19–48 years), and 20 (91%) were women; 15 (68%) came from South America, 5 (22%) came from Africa, and 2 (9%) came from Europe. Nineteen (95%) of 20 persons had symptoms for >1 month preceding their first medical encounter. Approximately 27.2% had pulmonary manifestations only, 36.4% had extrapulmonary manifestations only, and the remaining 36.4% had both pulmonary and extrapulmonary manifestations. *Mycobacterium tuberculosis* was found in 11 of 14 with pulmonary involvement, and chest radiograph was normal in 5 (22%). When compared with patients from the general population, women were more numerous (91% vs. 30%), and extrapulmonary tuberculosis was more frequent among undocumented residents (72% vs. 34%). The time from first symptoms to first consultation was also longer when compared to the general population and the registered South American residents (5% vs. 30% and 40%, respectively, consulting in the first month; p = 0.008). In 4 (19%) patients, resistance to >1 antituberculosis drug was identified, with no multidrug resistance (defined as rifampicin) identified, a rate of resistance similar to that seen in their countries of origin but higher than the Swiss rate (6.3%) (*4*). All patients were treated with a 4-drug regimen (HRZE: H = isoniazid, R = rifampicin, Z = pyrazinamid, E = ethambutol) for 2 months, followed by a 2-drug therapy (HR) for 4 months. Eighteen (82%) patients adhered to the regimen, as determined by monthly medical interviews and urine isoniazid checks. Only the 4 remaining patients who missed more than one third of the appointments with 50% of negative urine checks, or who defaulted, were placed under directly observed therapy. Fifteen (68%) patients regularly attended their appointments until completion of treatment. Seven (32%) patients left Switzerland before the end of treatment, 2 of whom were deported.

Fourteen (64%) patients were hospitalized to initiate treatment. Four had health insurance; the other patients contracted a debt for hospitalization. The lack of insurance did not influence adherence to treatment negatively. However, as a consequence of tuberculosis, 8 (66%) lost their jobs.

Of 102 identified close contacts, 88 (87%) were evaluated by tuberculin skin testing. Chest x-ray was performed on 21 (24%) patients with a positive test (>10 mm induration), and isoniazid was prescribed prophylactically. No secondary case of active tuberculosis was identified.

Most undocumented immigrants with tuberculosis in Geneva are young South American or African women engaged in domestic activities. This finding reflects the irregular work opportunities in Geneva, an area with little agriculture and industry. As suspected, a delay of several weeks occurred before seeking care (*5*). The economic and social impact of tuberculosis was high for this population. Two thirds of these patients lost their jobs as a consequence of tuberculosis. Joblessness could be an additional factor to further deter patients from seeking care. Adherence to treatment was good, which suggested confidence that care providers would not report to immigration authorities and that supportive follow-up care was available. Of more concern, approximately one third of the patients left Switzerland before completing the full course of treatment. This transfer rate (43%) of undocumented migrants corresponds to that observed among foreign-born patients with unknown legal status in Switzerland (*6*). Failure to complete a full course of treatment may lead to relapse and emergence of resistant strains. A growing proportion of cases of tuberculosis observed in Europe is in migrants, some undocumented, from the developing world (*3*). Strong political measures should be enforced to ensure access to healthcare services with respect to confidentiality (as recently stated in the Netherlands) (*7*). Much emphasis has been put on screening at time of arrival. Screening can be conducted for immigrants and asylum seekers, but undocumented migrants are not screened (*8*). Facilitated access to medical services and free affordable therapy is a necessity; active tuberculosis develops in most foreign-born residents several years after their arrival (*2*). In an era of high mobility, specific innovative programs should be established to control and prevent tuberculosis for this high-risk, foreign-born population. Early detection with nonidentifying tuberculosis tracking systems (*9*), screening at unspecialized clinics (*10*), and free treatment with adequate administrative measures are needed. Industrialized countries must take responsibility to reduce the spread of resistant tuberculosis.
